# Sweet Cherry Extract as Permeation Enhancer of Tyrosine Kinase Inhibitors: A Promising Prospective for Future Oral Anticancer Therapies

**DOI:** 10.3390/ph16111527

**Published:** 2023-10-27

**Authors:** Federica Poggialini, Chiara Vagaggini, Annalaura Brai, Claudia Pasqualini, Anna Carbone, Francesca Musumeci, Silvia Schenone, Elena Dreassi

**Affiliations:** 1 Department of Biotechnology, Chemistry and Pharmacy (DBCF), University of Siena, 53100 Siena, Italy; federic.poggialini@unisi.it (F.P.); chiara.vagaggini@student.unisi.it (C.V.); annalaura.brai@unisi.it (A.B.); pasqualini5@student.unisi.it (C.P.); 2 Department of Pharmacy, University of Genoa, 16132 Genoa, Italy; anna.carbone1@unige.it (A.C.); francesca.musumeci@unige.it (F.M.); silvia.schenone@unige.it (S.S.)

**Keywords:** tyrosine kinase, pyrazolo[3,4-*d*]pyrimidines, cancer, oral drug delivery, permeation enhancers, nutraceuticals

## Abstract

Although patients would rather oral therapies to injections, the gastrointestinal tract’s low permeability makes this method limiting for most compounds, including anticancer drugs. Due to their low bioavailability, oral antitumor therapies suffer from significant variability in pharmacokinetics and efficacy. The improvement of their pharmacokinetic profiles can be achieved by a new approach: the use of natural extracts enriched with polyphenolic compounds that act as intestinal permeability enhancers. Here, we propose a safe sweet cherry extract capable of enhancing oral absorption. The extract was characterized by the HPLC-UV/MS method, evaluated for in vitro antioxidant activity, safety on the Caco-2 cell line, and as a potential permeation enhancer. The sweet cherry extract showed a high antioxidant capacity (ABTS and DPPH assays were 211.74 and 48.65 µmol of Trolox equivalent/g dried extract, respectively), high content of polyphenols (8.44 mg of gallic acid per gram of dry extract), and anthocyanins (1.80 mg of cyanidin-3-glucoside equivalent per g of dry extract), reassuring safety profile (cell viability never lower than 98%), and a significant and fully reversible ability to alter the integrity of the Caco-2 monolayer (+81.5% of Lucifer yellow permeability after 2 h). Furthermore, the ability of the sweet cherry extract to improve the permeability (P_app_) and modify the efflux ratio (ER) of reference compounds (atenolol, propranolol, and dasatinib) and selected pyrazolo[3,4-*d*]pyrimidine derivatives was investigated. The obtained results show a significant increase in apparent permeability across the Caco-2 monolayer (tripled and quadrupled in most cases), and an interesting decrease in efflux ratio when compounds were co-incubated with sweet cherry extract.

## 1. Introduction

Many patients receive daily or weekly injections of single-dose therapeutics (such as vaccines), chronic therapies (e.g., insulin), or anti-cancer treatments including chemotherapeutics, such as bortezomib, or monoclonal antibody medication, such as daratumumab. Unfortunately, the patients’ non-adherence due to needle phobia, pain, and inconvenience of injections leads to missed therapeutic appointments and worse disease outcomes [1]. Due to its convenience, affordability, and high patient acceptance, oral therapy is the most preferred route of drug administration, but the perfect balance between aqueous solubility, membrane permeability, and chemical and enzymatic stability represents a challenging issue. Indeed, anticancer drugs are characterized by poor oral bioavailability linked to both suboptimal physicochemical properties and physiological barriers, which reduce the absorption [2]. As a result, anticancer drugs are traditionally administered intravenously, leading to severe side effects and the need of hospitalization and nursing cares. Furthermore, oral administration of anticancer treatments primarily reduces overall treatment costs and helps improve patients’ quality of life [3]. In recent decades, several strategies were adopted to overcome problems of bioavailability of oral anticancer therapies through the development of nanocarrier-based drug delivery systems, pharmacokinetic boosting (acting through the inhibition of physiological barriers via a drug-drug interaction), or intestinal permeation enhancers. To date, the majority of permeation enhancers (PEs) in clinical trials are chemical molecules including medium-chain fatty acids (MCFAs) such as sodium caprylate (C8), sodium caprate (C10), and sodium salcaprozate (SNAC), piperazine derivatives, and melittin, a 26-amino acid peptide derived from honeybee venom [1,4]. Although these PEs promote the absorption by the widening of tight junctions, they are commonly associated with cytotoxicity and physical damage to the intestinal membranes [5]. What if a revolutionary approach came from food, and more specifically, from plant foods? Epidemiological studies showed that food is a very powerful tool for maintaining wellbeing and preventing disease. In fact, nutrition is linked to many degenerative, autoimmune, and neoplastic diseases. Nutraceuticals are food components, extracts, or food derivatives that have potential health benefits beyond their nutritional value. Phytochemicals, such as polyphenols, are non-essential nutrients produced primarily by plants with defensive or disease-protective properties. In human health, they have specific pharmacological effects as antioxidant, anti-inflammatory, hepato and neuroprotective, anti-aging in DNA damage, and anti-cancer agents [6]. Indeed, enthusiasm for phenolic-rich materials grew significantly over the past few years due to their ability to delay the onset of chronic diseases, including cancer. According to the literature, Lamson et al. already identified a wide range of polyphenol-rich plant foods with permeation enhancer properties; in particular, they found out that pelargonidin, a strawberry-derived permeation enhancer, enables oral insulin delivery [1]. In addition, new research showed that phenolic compounds may act as P-glycoprotein (P-gp) inhibitors, preventing multidrug resistance and improving the therapeutic efficacy of drugs. Alves et al. demonstrated that polyphenol-enriched sweet cherry fractions may effectively inhibit P-gp activity on the MDCK-MDR1 cell line [7]. Based on these concerns and strongly believing in the therapeutic potential of food extracts, we decided to investigate the ability of sweet cherry extract to act as a permeation enhancer. With the aim of investigating the ability of sweet cherry extract (SCE) to increase the permeability across the Caco-2 monolayer and modify consequently the efflux ratio, we selected three reference compounds according to their oral bioavailability data known from the literature: atenolol as a low permeable compound, absorbed via passive paracellular transport and characterized by a low percentage of oral bioavailability (around 50%), propranolol selected as the high permeable probe absorbed almost around 90% of the oral dose via passive transcellular mechanisms, and dasatinib selected as a reference orally administrated tyrosine kinase inhibitor (TKI). We also investigated the permeation-enhancing effect of cherry extract with selected in-house pyrazolo[3,4-*d*]pyrimidines. These derivatives, known to be potent tyrosine kinase inhibitors active against both solid and liquid tumors (e.g., glioblastoma, neuroblastoma, and leukaemia), were selected on the basis of previously published in vitro ADME data [8,9,10]. Since our work would concern the possible alterations of permeability in the presence of the cherry extract, pyrazolo-pyrimidine derivatives were heterogeneously selected according to this property. Indeed, as reported in Table 1, Cpd **1** and **5** were characterized by the higher PAMPA P_app_ values over 10 × 10^−6^ cm/s, Cpds **2**,**3**,**7** were endowed with apparent permeability between 3.2 and 5.5 × 10^−6^ cm/s, while Cpd **4** and **6** resulted in the permeable derivatives of the series with P_app_ minor lower than 1 × 10^−6^ cm/s. Despite their promising anticancer activity, pyrazolo[3,4-*d*]pyrimidines are characterized by a low aqueous solubility profile, which may reduce their in vivo bioavailability depending on the solvation rate, a generally good ability to cross phospholipidic bilayers exploiting passive mechanisms without remaining entrapped into the phospholipidic bilayer, and a high metabolic stability when incubated in the presence of human liver microsomes.

Before starting with the permeability experiments, the sweet cherry extract was characterized by the HPLC-UV/MS method to identify the most abundant polyphenolic compounds, by quantifying the total phenolic content (TPC), the anthocyanins content (TAC), and analyzing the antioxidant activity recurring to DPPH and ABTS assays. Having established the non-toxicity of the extract on Caco-2 cells, we carried out cytotoxicity studies to identify the most tolerable concentration of compounds to be used in the permeability studies. We then proceeded to evaluate the effective ability of the cherry extract to increase the permeation of Lucifer yellow through the Caco-2 monolayer, and the subsequent ability of the monolayer to restore the initial conditions after removal of the extract. The fivefold increase in Lucifer yellow permeability in the presence of the sweet cherry extract (SCE) led us to perform further permeability studies by incubating reference and pyrazolo-pyrimidine compounds in the absence and presence of 1 mg/mL of SCE. Finally, to ensure that the treatment with SCE did not induce the formation of openings large enough to allow the passage of large materials, the cellular monolayer was incubated with the macromolecular tracer human serum albumin (HSA) with and without SCE. Although further investigations are required to fully characterize the extract, identify the bioactive fraction, and investigate the mechanism of action in more detail, the high safety profile, and the significant and reversible increase in permeability of SCE led us to hypothesize the development of new oral therapeutic approaches to improve treatments and facilitate the oral administration of a wide range of injection therapies.

## 2. Results and Discussion

### 2.1. Sweet Cherry Extract: Yield and HPLC-UV/MS Characterization

**Figure 1:**
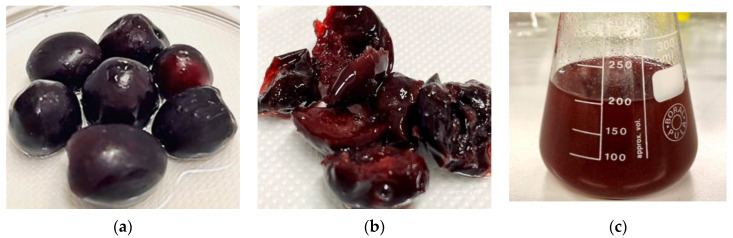
Pictures illustrate sweet cherries during the process of extraction. Sweet cherries were (**a**) deprived of stems and washed with H_2_O-MillQ, then (**b**) pits were removed and cherries weighed; lastly the extraction process started (**c**) with the homogenization of fruit materials in a mixture of H_2_O/EtOH 1:1 *v*/*v*.

The primary goal of extraction was to maximize yield while reducing the amount of unnecessary compounds, such as sugars or proteins, which might cause problems with the stability and quality of the extract. Many variables can affect the success of the process, including solvent, the mass-to-solvent ratio, temperature, and pH. An H_2_O/EtOH (1:1 *v*/*v*) mixture was chosen as the extraction mixture because (1) it is commonly used as a solvent mixture to obtain an extract enriched in bioactive compounds, (2) in a perspective of recycling and green chemistry, both solvents are sustainable and can be reused during the process, and (3) in a future purpose of in vivo evaluation, the use of food-grade solvents ensures that any residue will not be toxic after ingestion. As a first step, the cherries were carefully prepared for the extraction process; as shown in Figure 1, the fruits were washed with water, and after removing pits, weighted and homogenized with a hydroalcoholic (H_2_O/EtOH 1:1 *v*/*v*) solution.

For the preparation of the extract, we applied a conventional extraction procedure carried out at room temperature (RT) without resorting to microwaves, ultrasounds, or high temperature methodologies. The efficacy of the extraction process was reported as percentage of yield, which resulted in 15.38 ± 0.93%, a value in agreement with data previously reported in the literature when applying a classical extraction with low temperature for the preparation of the cherry extract [11]. The sweet cherry extract was stored in the dark at −20 °C to avoid degradation processes. The SCE was first characterized by the HPLC-UV/MS method to identify the most abundant components. For this purpose, 50 mg of SCE were weighed and resuspended in 1 mL of H_2_O^+^/ACN^+^/MeOH^+^ mix (1:1:1 *v*/*v*), acidified with 0.1% *v*/*v* of formic acid (FA), were filtered using a 0.45 µm filter (purchased by Acrodisc), and analyzed by the chromatographic method described below (Materials and Methods chapter). As reported in Table 2 (see also Figure S1 in the Supplementary Materials), several flavonoids and chlorogenic acid derivatives were identified from the chromatographic analysis, as well as anthocyanins, such as cyanidin-3-O-rutinoside, pelargonidin-3-O-rutinoside, and peonidin-3-O-rutinoside, which are responsible for the red and bright skin color, as well as the flavonols such as quercetin-3-O-rutinoside, quercetin-3-O-rutinoside-7-O-glucoside, quercetin-3-O-galactosyl-rhamnoside, and kaempferol-3-O-rutinoside, which are commonly found in high amounts in cherry pulp [12]. Finally, the HPLC-UV-MS analysis highlighted the presence of some chlorogenic acid derivatives. The most abundant of these was *trans*-3-O-caffeoylquinic acid followed by *trans,trans*-3,5-di-O-caffeoylquinic acid.

### 2.2. Total Phenolic Content (TPC), Antioxidant Activity and Total Anthocyanins Content (TAC)

Cherry extract was analyzed in terms of antioxidant activity, total phenols, and anthocyanins content. The nutraceutical properties of polyphenols are related to their antioxidant and metal-chelating capability. In addition, polyphenols can interact with enzymes and receptors, modulating cellular pathways [13]. Regular phenolic intake helps to prevent several diseases caused by stress and oxidative stress, including cardiovascular disease, diabetes, neurological disorders, and cancer [13,14,15]. As reported in Table 3, TPC in the cherry extract is 8.44 mg GAE/g DE, a value in agreement with data previously reported for sweet cherry cultivars after conventional extraction, but lower than those obtained after microwave-assisted extraction [11,16,17].

TPC in cherries is valuable, higher than those reported for bananas, melon, apples, and oranges [18]. Anthocyanins are a subclass of polyphenols and water-soluble pigments, responsible for the red color of cherries and are mainly contained in skins [16]. Interestingly, the quantity of anthocyanins increases during the fruit ripening process and is closely related to cultivars [19,20]. Due to the presence of many hydroxyl groups, anthocyanins have a higher antioxidant activity compared to other classes of natural antioxidants [16]. Grapes and berries are rich in anthocyanins, in particular, raspberries, wild blueberries, cherries, black and red currants, strawberries, and pomegranates. TAC of the cherry extract is 1.80 mg Cy-3-glu/g DE, a value in agreement with those already reported for sweet cherries [11,20,21]. The antioxidant activity determined with ABTS and DPPH assays is 211.74 and 48.65 µmol of TE/g DE, respectively. Even in this case, results are in the range reported for sweet cherries in previously published papers and confirm the high nutraceutical value of cherries [19,22].

### 2.3. Cell Viability Studies

**Figure 2:**
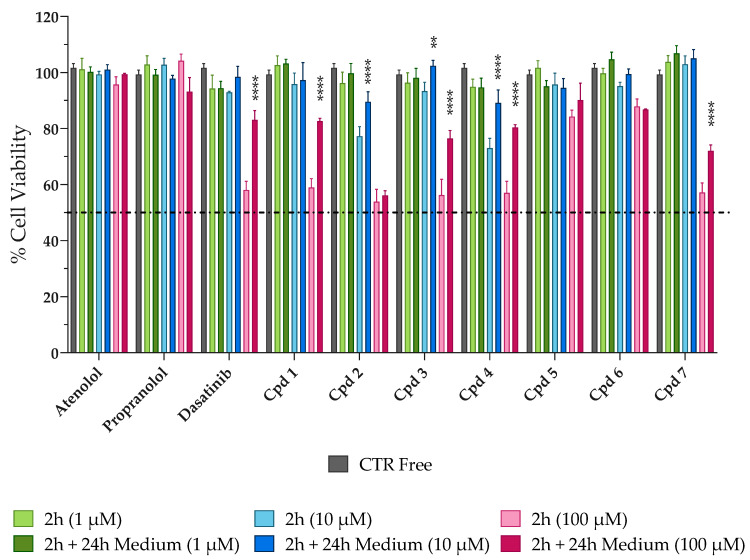
Evaluation of the potential cytotoxic effect of the reference and tested compounds on Caco-2 cells. Cells were exposed to increasing concentrations (1–10–100 µM; green, blue, and pink columns, respectively) of tested compounds for 2 h (the lighter columns) and then for further 24 h in fresh, free-drug medium (the darkest columns). CTR Free represents the control untreated. MTT assay was used to evaluate the cell viability. Dasatinib **** *p*< 0.0001 2 h (100 µM) vs. 2 h + 24 h Medium (100 µM), Cpd **1** **** *p*< 0.0001 2 h (100 µM) vs. 2 h + 24 h Medium (100 µM), Cpd **2** **** *p* < 0.0001 2 h (10 µM) vs. 2 h + 24 h Medium (10 µM), Cpd **3** ** *p* = 0.0049 2 h (10 µM) vs. 2 h + 24 h Medium (10 µM) and **** *p* < 0.0001 2 h (100 µM) vs. 2 h + 24 h Medium (100 µM), Cpd **4** **** *p* < 0.0001 2 h (10 µM) vs. 2 h + 24 h Medium (10 µM) and **** *p* < 0.0001 2 h (100 µM) vs. 2 h + 24 h Medium (100 µM), and Cpd **7** **** *p* < 0.0001 2 h (100 µM) vs. 2 h + 24 h Medium (100 µM) (two-way ANOVA followed by Tukey’s multiple comparisons test).

Before conducting permeability studies, SCE and tested compounds were evaluated in terms of cytotoxicity on Caco-2 cells. Indeed, it was important to ensure that the increase in permeability across the cellular monolayer was due to the effect of SCE on multi-protein complexes (e.g., tight junctions, adherens junctions, and desmosomes) and not to the toxic effect of both SCE and compounds. When Caco-2 cells were exposed to increasing concentrations of SCE (0.25–1 mg/mL), no significant loss in cell viability was detected both after 2 h of treatment and 2 h followed by 24 h of incubation in free medium as shown in Figures S2 and S3 (Supplementary Materials). Since no significant changes were detected among the tested concentrations of SCE, we selected 1 mg/mL for further permeability experiments. The same aspect was investigated for tested compounds. As shown in Figure 2, when Caco-2 cells were incubated in the presence of increasing concentrations (1–10–100 µM) of reference compounds (atenolol, propranolol, and dasatinib) and pyrazolo[3,4-*d*]pyrimidine derivatives for 2 h, no significant signs of toxicity were detected for the lowest concentration of 1 µM [2 h (1 µM), light green column] compared to the control (CTR Free, grey column).

In addition, the mid-concentration [2 h (10 µM), light blue column] was well tolerated by the cells with percentages of cellular viability never lower than 70%. As expected, when cells were exposed to the highest concentration [2 h (100 µM), light pink column], albeit for a short period of time of 2 h, cell viability decreased in most cases at 50–60%, with the exception of atenolol and propranolol, which maintained high percentages of viable cells, confirming the good safety profile on Caco-2 cells. To verify whether the decrease in cell viability could be reversible or irreversible and to better select the most appropriate concentration for permeability studies, the cells were washed with PBS after 2 h of treatment and incubated with fresh medium for another 24 h [2 h + 24 h darker green (1 µM), blue (10 µM), pink (100 µM) columns, respectively]. As shown in Figure 2, all compounds induced a reversible cytotoxic effect on Caco-2 cells. In fact, when looking at the darkest columns, the percentage of viable cells never decreased, remaining constant or increasing after 2 h + 24 h of incubation with fresh medium. These results are significantly evident for dasatinib and the pyrazolo[3,4-*d*]pyrimidine compounds with percentages of cell viability restored to around 90–100% for 10 µM and 70–80% for 100 µM. Considering the cytotoxicity data obtained, we selected the intermediate concentration of 10 µM for further permeability studies, as it was better tolerated by Caco-2 cells (more than 70% of cell viability after 2 h) than 100 µM and because it would give a better chromatographic response than 1 µM.

### 2.4. Permeability Studies

**Figure 3:**
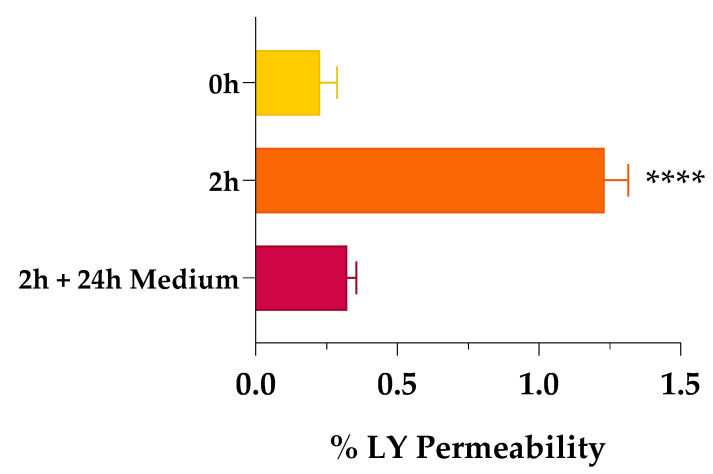
Percentage of Lucifer yellow (LY) permeability across Caco-2 monolayer. Percentage of LY permeability was monitored at time 0 (0 h, yellow bar), after 2 h of treatment (2 h, orange bar), and after 24 h of fresh, SCE-free medium incubation (2 h + 24 h Medium, red bar). Values are the means ± SD of *n* = 3 independent experiments run in triplicate. **** *p* < 0.0001 2 h vs. 0 h (one-way ANOVA followed by Dunnett’s multiple comparisons test).

When we assessed the non-cytotoxicity of SCE, we investigated the potential permeation enhancer efficacy of the extract by tracing the passage of the impermeable, fluorescent dye Lucifer yellow (LY) across the Caco-2 cell monolayer. As shown in Figure 3, when the Caco-2 monolayer was exposed to 1 mg/mL of SCE dissolved in HBSS, the percentage of LY permeability (% LY P_app_) increased significantly after 2 h of treatment (% LY P_app_ of 1.233 ± 0.083, orange bar) compared to the beginning of the experiment (% LY P_app_ of 0.228 ± 0.060, yellow bar). Indeed, the amount of LY passed through the monolayer was increased more than five-fold. As reversibility is a crucial feature of a safe permeation enhancer, the passage of LY across the Caco-2 monolayer was also monitored 24 h after removal of the sweet cherry extract. Recovery at 24 h was almost complete (% LY P_app_ of 0.322 ± 0.033, red bar), with a four-fold reduction in the percentage of LY permeability compared to 2 h (orange bar). These encouraging results led us to rule out some phenomena of over-permeabilization, which in vivo could lead to physiopathological changes induced by inflammation.

**Figure 4:**
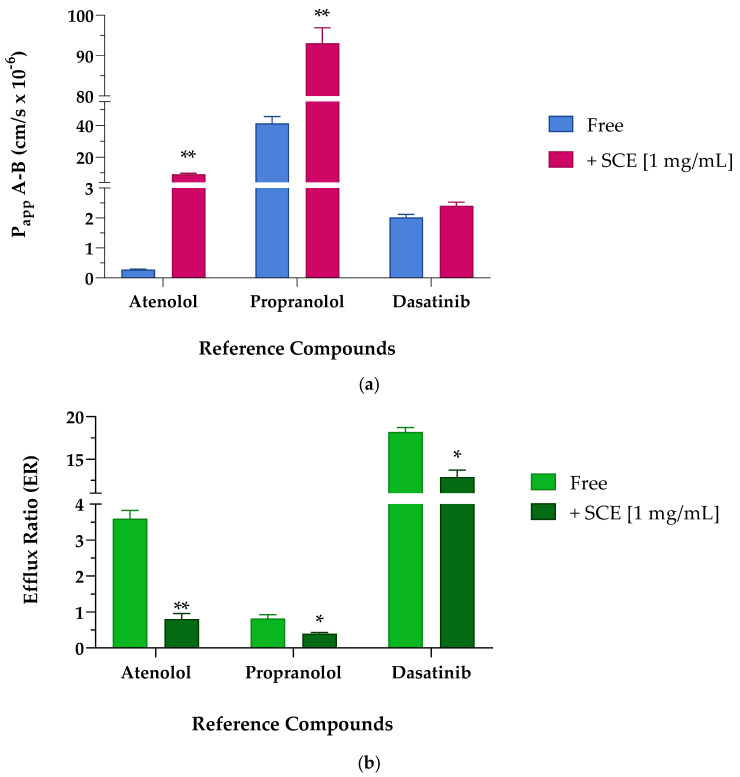
Apparent permeability (P_app_ cm/s × 10^−6^) values (**a**) and efflux ratio (ER) (**b**) across Caco-2 cellular monolayer of reference compounds atenolol, propranolol, and dasatinib. (**a**) Apparent permeability (P_app_) values are reported as mean ± S.D. of several time points (0.25–0.5–1–2 h). Each experiment was run in triplicate (*n* = 3). Caco-2 cells were treated with 10 µM of reference compounds in the absence (Free, blue bar) and presence (+SCE [1 mg/mL], pink bar) of sweet cherry extract. Atenolol ** *p* = 0.0021 Free vs. +SCE [1 mg/mL], propranolol ** *p* = 0.0061 Free vs. +SCE [1 mg/mL], (unpaired Student *t*-test). (**b**) Efflux ratio values are reported as mean ± S.D. of several time points (0.25–0.5–1–2 h). Each experiment was run in triplicate (*n* = 3). Caco-2 cells were treated with 10 µM of reference compounds in the absence (Free, light green bar) and presence (+SCE [1 mg/mL], dark green bar) of sweet cherry extract. Atenolol ** *p* = 0.0049 Free vs. +SCE [1 mg/mL], propranolol * *p* = 0.0346 Free vs. +SCE [1 mg/mL], and dasatinib * *p* = 0.0157 Free vs. +SCE [1 mg/mL] (unpaired Student *t*-test).

To evaluate the potential ability of SCE to act as a permeation enhancer, we studied the effect on the apparent permeability of reference compounds, such as atenolol, propranolol, and dasatinib, whose permeability and efflux ratio data, as well as the oral absorption, are known from literature. Atenolol is known to be a molecule unable to cross membranes except by passive paracellular transport (P_app_ less than 1 × 10^−6^ cm/s), as confirmed by its poor oral adsorption (approximately 50% of an oral dose). Propranolol, on the other hand, is characterized by P_app_ values higher than 10 × 10^−6^ cm/s, suggesting its efficiency in crossing membranes and distributing in tissues, as reflected by almost 90% of oral adsorption [23,24]. Dasatinib was chosen as a reference compound as it is an oral TKI clinically used in the treatment of liquid tumors such as leukaemia. According to the European Medicines Agency’s (EMA) reports, dasatinib was classified as a BCS class II compound endowed with a bioavailability ranging from 14 to 34% in animals, and 15.2% in humans [25]. As reported in Table 4 and shown in Figure 4a, the apparent permeability from the apical to the basolateral compartment (P_app_ A-B) of the reference compounds improved when co-incubated in the presence of 1 mg/mL of SCE. The low permeable marker, atenolol, significantly increased its P_app_ by about 30-fold in the presence of the extract. The high permeable probe propranolol as well, significantly doubled its ability to cross Caco-2 monolayer. Albeit to a smaller and less significant extent, also the P_app_ value of dasatinib underwent a slight increase when co-incubated with SCE, probably due to the inhibitory action of the extract on P-gp [7]. Since dasatinib is a known substrate of P-gp, the suboptimal permeability across the cellular monolayer both in the absence and presence of the sweet cherry extract did not surprise us because the greater amount of compound passing through the basolateral compartment was repumped in the apical one [26,27]. Regarding the secretory direction, the permeability from the basolateral to apical compartment (P_app_ B-A) of propranolol and dasatinib did not show relevant changes between Free and + 1 mg/mL of SCE. Atenolol, on the other hand, underwent a more interesting seven-fold implementation of P_app_ B-A when in the presence of SCE.

The apparent permeability secretory data (P_app_ B-A) results are particularly relevant as the index of the efflux ratio. The ratio of the two P_app_ coefficients (P_app_ B-A/P_app_ A-B), defined as the efflux ratio (ER), can be the first indication of the involvement of active transport mechanisms in the absorption process. For this reason, it is always preferable to perform transport experiments across the Caco-2 monolayer in both the absorptive and secretory directions. From a first look at the apparent permeability values in both directions and as better reported in Table 5 and Figure 4b, the presence of sweet cherry extract allowed a reduced efflux, particularly evident for atenolol and dasatinib. According to the literature, the polarized transport of atenolol across Caco-2 cells resulted in an efflux ratio greater than 2, suggesting that it may be subject to active efflux mechanisms [28].

Atenolol, reported to be a P-gp substrate [29], when incubated alone showed an efflux ratio value greater than 3, in agreement with results reported by other laboratories [30]. Indeed, studies on the bioavailability of atenolol confirmed that transporter-mediated efflux may play a key role in the transport of atenolol across membranes [30]. When the experiment was repeated in the presence of the cherry extract, the efflux ratio was significantly reduced by more than four times, confirming the potential inhibitory effect on P-gp. Propranolol, a non-P-gp substrate, showed an efflux ratio of less than 2, confirming that it is not subject to active transport mechanisms [31]. Nevertheless, the co-incubation of propranolol with SCE almost halved the ER. Dasatinib underwent massive efflux mechanisms and showed the highest ER value among the studied reference compounds. Considering the pharmacokinetic parameters reported in the literature and the high affinity towards P-gp, a significantly increased efflux ratio was expected [24,32]. As seen for atenolol, also the efflux ratio of dasatinib was reduced in the presence of sweet cherry extract, probably due to the inhibition of the efflux pumps’ activity.

**Figure 5:**
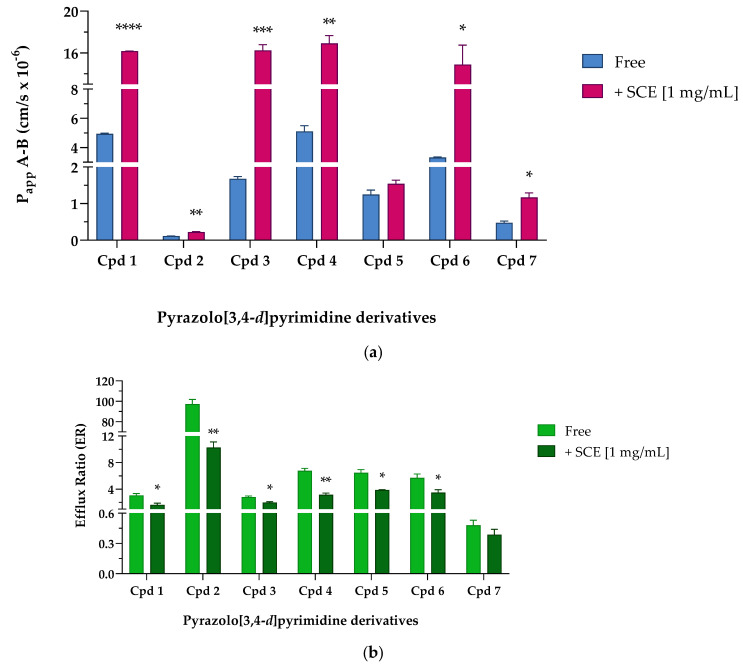
Apparent permeability from the apical to basolateral compartment (P_app_ A-B × 10^−6^ cm/s) values (**a**) and efflux ratio (**b**) across Caco-2 cellular monolayer of seven pyrazolo[3,4-*d*]pyrimidine derivatives. (**a**) Apparent permeability (P_app_) values are reported as mean ± S.D. of different time points assayed (0.25–0.5–1–2 h). Each experiment was run in triplicate (*n* = 3). Caco-2 cells were treated with 10 µM of tested compounds in the absence (Free, blue bar) and presence (+SCE [1 mg/mL], pink bar) of sweet cherry extract. Cpd **1** **** *p* < 0.0001 Free vs. +SCE [1 mg/mL], Cpd **2** ** *p* = 0.0037 Free vs. +SCE [1 mg/mL], Cpd **3** *** *p* = 0.0007 Free vs. +SCE [1 mg/mL], Cpd **4** ** *p* = 0.0025 Free vs. +SCE [1 mg/mL], Cpd **6** * *p* = 0.0128 Free vs. +SCE [1 mg/mL], and Cpd **7** * *p* = 0.0170 Free vs. +SCE [1 mg/mL] (unpaired Student *t*-test). (**b**) Efflux ratio data are reported as mean ± S.D. of several time points tested (0.25–0.5–1–2 h). Each experiment was run in triplicate (*n* = 3). Caco-2 cells were treated with 10 µM of reference compounds in the absence (Free, light green bar) and presence (+SCE [1 mg/mL], dark green bar) of sweet cherry extract. Cpd **1** * *p* = 0.0329 Free vs. +SCE [1 mg/mL], Cpd **2** ** *p* = 0.0014 Free vs. +SCE [1 mg/mL], Cpd **3** * *p* = 0.0271 Free vs. +SCE [1 mg/mL], Cpd **4** ** *p* = 0.0069 Free vs. +SCE [1 mg/mL], Cpd **5** * *p* = 0.0175 Free vs. +SCE [1 mg/mL], and Cpd **6** * *p* = 0.0498 Free vs. +SCE [1 mg/mL] (unpaired Student *t*-test).

After fine-tuning the protocol, we proceeded to study the potential permeability-enhancing effect of sweet cherry extract on pyrazolo[3,4-*d*]pyrimidine derivatives selected from our in-house large library. Table 6 shows the P_app_ A-B and P_app_ B-A values obtained in the absence (Free) and presence (+SCE [1 mg/mL]) of cherry extract (see also Figure 5a).

When pyrazolo[3,4-*d*]pyrimidine derivatives were incubated without the extract, Cpd **1**, **4**, and **6** showed the highest permeability. Cpd **1**, whose PAMPA permeability was one of the highest among the derivatives (see Table 1), showed an equally good ability to cross more complex systems, such as a cellular monolayer. According to the passive permeability obtained from PAMPA assay (see Table 1), Cpd **4** and **6** were not classified as compounds able to efficiently cross a phospholipid bilayer, whereas their permeability improved in the presence of Caco-2 cells. These results led us to hypothesize the central role of influx pumps such as organic cation transporters (OCTs), organic-anion-transporting polypeptides (OATPs), or ileal apical sodium/bile acid co-transporters (ASBTs) in promoting the active passage of Cpd **4** and **6** across membranes [24,33]. If Cpd **3** confirmed to be moderately able to cross membranes both passively and actively (PAMPA P_app_ reported in Table 1, Caco-2 P_app_ A-B 1.680 ± 0.061 cm/s × 10^−6^), Cpd **5** reached the intracellular compartment throughout passive diffusion rather than by exploiting active influx systems; indeed, Caco-2 P_app_ A-B of Cpd **5** resulted almost 10-fold lower than PAMPA P_app_. Finally, when incubated without sweet cherry extract, Cpd **2** and Cpd **7** showed suboptimal P_app_ A-B values of less than 0.5 × 10^−6^ cm/s. Indeed, if PAMPA data (Table 1) suggested that both compounds were moderately permeable exploiting passive diffusion mechanisms, Caco-2 permeability studies indicated that Cpd **2** and **7** were unable to efficiently cross a cellular monolayer. Then, pyrazolo[3,4-*d*]pyrimidine compounds were co-incubated in the presence of 1 mg/mL of sweet cherry extract; as shown in Figure 5a (pink column), the transport of pyrazolo[3,4-*d*]pyrimidine derivatives across the Caco-2 monolayer were generally increased in the presence of SCE. Cpd **1**, **4**, and **6** significantly improved the permeability in the absorptive direction (P_app_ A-B) by about three and four times, respectively. Moreover, Cpd **3** showed the same trend, significantly improving the P_app_ by almost 10-fold. Considering Cpd **5**, the co-presence of the sweet cherry extract induced only a slight improvement in the P_app_ A-B values, while Cpd **2** and **7** almost doubled the value of P_app_ A-B with respect to the Free condition. In the secretory direction, the presence of the SCE induced only a slight increase in the apparent permeability of Cpd **7**, while Cpd **2** and **5** were characterized by a significant reduction in P_app_ B-A when in the presence of sweet cherry extract. Compounds **1**, **3**, **4**, and **6**, on the other hand, underwent a more interesting implementation of P_app_ B-A. As already mentioned, the permeability data in the secretory direction (P_app_ B-A) were particularly useful for the evaluation of the efflux ratio (ER). Table 7 and Figure 5b show the ER values obtained when pyrazolo-pyrimidine derivatives were incubated Free and with 1 mg/mL of SCE. It was reported in the literature that TKIs interact with efflux transporters both as substrates and as inhibitors [34,35,36]. Indeed, TKIs that are substrates for efflux transporters can sometimes act as transporter inhibitors when used at high concentrations [36]. In our free condition experiments, pyrazolo[3,4-*d*]pyrimidine derivatives were characterized by ER values greater than 2, suggesting the possibility of being subject to active transport mechanisms made by P-gp or other ATP-binding cassette (ABC) transporters. In detail, Cpd **1** and **3** may be identified as weak substrates thanks to their ER values around 3, while Cpd **4**, **5**, and **6** showed ER values between 5.708 and 6.769, suggesting a greater interaction with active transports.

**Figure 6:**
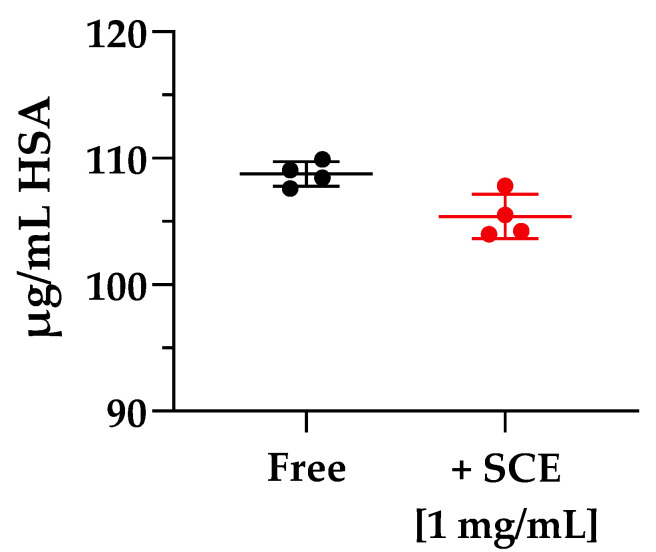
Molecule cargoes’ size experiment across Caco-2 monolayer. The experiment ran for 2 h in the absence (Free, black) and in the presence (+SCE [1 mg/mL], red) of sweet cherry extract. Error bars represent S.D. (*n* = 4).

The two diametrically opposed borderline cases are represented by Cpd **2** and **7**; in fact, if the first one (Cpd **2**) showed the highest ER value of the series (97.256 ± 4.489) due to a strong interaction with P-gp or other active transport pumps, Cpd **7** showed the lowest efflux ratio among the pyrazolo-pyrimidine derivatives, 0.517 ± 0.014, ensuring that this derivative was not subject to active efflux mechanisms. When incubated in the presence of the sweet cherry extract, a general and constant reduction in the ER values was observed; most of them (Cpds **1**, **3**, **4**, **5**, and **6**) were almost halved, and Cpd **7** showed only a slight decrease in the ER value, while Cpd **2** underwent a significant nine-fold reduction in the efflux ratio. Finally, experiments focusing on the size of molecules transported across the cellular monolayer were needed to ensure that the treatment with sweet cherry extract did not result in too large a gap that allowed the passage of pathogens or xenobiotics harmful to the organism. Lamson et al. demonstrated that the improved uptake of orally administrated 40-, 70-, and 150-kDa dextrans decreased with increasing cargo size when incubated in the presence of the anthocyanin pelargonidin extracted from strawberries [1]. Their findings suggest that pelargonidin did not allow large material, such as virus capsids or bacteria, to migrate out of the intestines and into the body. Based on these results, we decided to perform an analogue experiment exploiting the large molecular size of human serum albumin (HSA, 66.4 kDa). As shown in Figure 6, when Caco-2 cells were incubated with 1 mg/mL of HSA in HBSS in Free conditions, only a small amount of HSA (108.760 ± 0.978 µg/mL of HAS, Free), about one-tenth of the initial concentration was detected in the basolateral compartment after 2 h of treatment.

In the presence of 1 mg/mL of sweet cherry extract, no relevant changes in the amount of HSA passed through the cellular membrane were detected (104.887 ± 2.440 µg/mL of HAS, +SCE [1 mg/mL]). These results are particularly encouraging, as they demonstrate that the permeation-enhancing activity of SCE did not over-permeabilize the Caco-2 monolayer to allow passage of large cargoes. Although further in vivo experiments are required to fully explain the potential of the sweet cherry extract as a permeation enhancer and establish its long-term tolerability, it is possible to state that sweet cherry extract showed great potential as a nutraceutical source of polyphenolic compounds endowed with reversible permeation-enhancing activity and no toxic effects.

## 3. Materials and Methods

### 3.1. Compounds and Chemicals

All reagents, solvents, and drugs such as atenolol and propranolol were purchased from Sigma Aldrich S.r.l. (Milan, Italy). Dasatinib was supplied by Enamine (Kyiv, Ukraine). Cell culture media, buffers, and supplements, including fetal bovine serum (FBS), L-glutamine, and penicillin–streptomycin, were purchased from Euroclone S.p.A. (Milan, Italy). The human colorectal adenocarcinoma Caco-2 cell line was kindly donated by Professor Maria Frosini from the Department of Life Sciences (University of Siena, Siena, Italy). Pyrazolo[3,4-*d*]pyrimidine derivatives used in this project were previously synthesized and reported by us [8,9,10]. The respective NMR characterization, chemical structures, and names are provided in the Supplementary Materials (Chemistry Section and Scheme S1). For all the compounds, a 20 mM dimethyl sulfoxide (DMSO) stock solution was prepared prior to use and diluted to final concentration with cell culture medium (%DMSO never exceeded 0.05% *v*/*v* of final volume).

### 3.2. Fruit Material

The samples of sweet cherries (*Prunus avium* L.) used in this work were harvested in June 2022 in the Sienese countryside, Italy (43°19′06″ N 11°19′53″ E). Fruits were collected randomly from three trees. Fruits were harvested according to the ripening stage of the sweet cherry and selected according to the dark color of the skin at stage 12 to ensure the highest amount of nutrients, phytochemical composition, and bioactive compounds (BCs). The cherries were preserved at −20 °C before the extraction and stored at 5 °C during the analyses.

### 3.3. Preparation of Extracts

Before the extraction procedure, cherries were washed in cold MillQ water, and stems and pits were removed by hand (Figure 1a). Accurately weighed 10 g of crushed cherries (Figure 1b) were homogenized with 30 mL of a 1:1 *v*/*v* of EtOH/H_2_O solution using a T10 basic ULTRA-TURRAX^®^ homogenizer (Bioclass, Pistoia, Italy) (Figure 1c). The extraction process was performed at room temperature (23 °C) by stirring for 1 h and sonicating the homogenized cherries for 30 min. The extract was centrifuged for 15 min at 5000 rpm and the supernatant collected in a volumetric flask. The extract was dried under a N_2_ flow. The extraction yield was calculated according to the following equation (Equation (1)):

Extraction yield (%) = (dried extract (g))/(fresh cherry (g)) × 100.(1)

### 3.4. Sweet Cherry Extract Characterization via HPLC-UV/MS Method

Chromatographic analyses of sweet cherry extract were performed using the Agilent 1100 LC/MSD VL system (G1946C) purchased from Agilent Technologies (Palo Alto, CA, USA). Separations were performed at room temperature (RT) using a reverse-phase LUNA C18 column (5 µm, 100 Å, 250 mm × 4.6 mm), purchased from Phenomenex (Torrance, CA, USA). The mobile phase consisted of H_2_O^+^ and ACN^+^/MeOH^+^ (1:1 *v*/*v*) both acidified with 0.1% *v*/*v* formic acid (FA). The elution gradient started with 100% H_2_O^+^ for 5 min, increased to 100% of ACN^+^/MeOH^+^ in 25 min, and remained stable for a further 3 min before returning to the initial conditions in 120 s. Analyses were conducted at a flow rate of 0.6 mL/min, and UV detection was monitored at 360 and 520 nm. Spectra were acquired in both positive and negative modes with a scan range of *m*/*z* 100–1500. The injection volume was 20 µL.

### 3.5. Quantification of Total Phenolic Content (TPC)

TPC of the cherry extract was determined as previously described [37]. In detail, to 100 µL of each diluted extract, 200 µL of the Folin reagent was added. Then, 1 mL of 10% w/w sodium carbonate aqueous solution was added, followed by 1.7 mL of water. The mixture was allowed to react for 30 min in the dark at room temperature. The absorbance was measured at 765 nm (UV/Visible Lambda 2 spectrophotometer, Perkin Elmer, Norwalk, CT, USA). The results are expressed as the mg equivalent of gallic acid (GA) per gram of dry extract (mg GAE/g DE).

### 3.6. In Vitro Antioxidant Activity

The antioxidant activity of the cherry extract was determined using two different methods: DPPH radical scavenging assay, and ABTS radical cation (ABTS^+^) decolorization assay.

#### 3.6.1. DPPH Assay

The radical scavenging activity was determined as previously reported [38], using Trolox as the standard compound. In detail, 100 µL of the cherry extract was added to 1.0 mL of 0.2 mM DPPH methanolic solution, followed by 1.9 mL of MeOH. The mixture was allowed to react for 30 min in the dark at room temperature. The absorbance was measured at 517 nm (U*v*/*v*isible Lambda 2 spectrophotometer, Perkin Elmer, Norwalk, CT, USA). A blank was taken with a mixture of methanol and cherry extract. The results are expressed as TEAC (µmol of Trolox equivalent per gram of dry extract).

#### 3.6.2. ABTS Method

The antioxidant activity of the cherry extract was determined as reported by Brai et al. with minor modifications [38], using Trolox as standard compound. In detail, 100 µL of cherry extract was added to 1.5 mL of ABTS solution followed by 1.4 mL of EtOH. The mixture was allowed to react for 10 min in the dark at room temperature. The absorbance was measured at 751 nm (U*v*/*v*isible Lambda 2 spectrophotometer, Perkin Elmer, Norwalk, CT, USA). A blank was taken with a mixture of ethanol and cherry extract. The results are expressed as TEAC (µmol of Trolox equivalent per gram of dry extract).

### 3.7. Quantification of Total Anthocyanins Content (TAC)

The total anthocyanins content (TAC) of the cherry extract was determined as described by Vilas-Boas et al. [11]. In detail, 200 µL each extract was mixed with 800 µL of KCl buffer at pH 1 or CH3COONa at pH 4.5. After 15 min of equilibration at room temperature, the absorbance was measured at 515 and 700 nm (U*v*/*v*isible Lambda 2 spectrophotometer, Perkin Elmer, Norwalk, CT, USA). The absorbance (A) of the sample was calculated according to Equation (2):

A = (Abs 515 nm − Abs 700 nm) pH 1.0 − (Abs 515 nm − Abs 700 nm) pH 4.5.(2)

The TAC, in mg/L, was calculated as follows:

C (mg/L) = (A × MW × DF × 1000)/ε × L(3)

where molecular weight (MW) and molar extinction coefficient (ε) of cyanidin-3-glucoside is 449.2 g/mol and 26,900 g/mol, respectively; cuvette length (L) is 1 cm, and final dilution factor (DF) is 10. Results are expressed as milligrams of cyanidin-3-glucoside equivalent per g of dry extract (mg Cy-3-glu/g DE).

### 3.8. Caco-2 Cell Culture for Viability Studies

Caco-2 cells (passage number 18–50) were cultured Dulbecco’s Modified Eagle Medium (DMEM) containing 20% fetal bovine serum (FBS), 2 mM L-glutamine, and 10.000 U/mL penicillin/streptomycin at 37 °C in a fully humidified 5% CO_2_ atmosphere. Cells were sub-cultured using a 1× trypsin/EDTA solution and then passed every 3–4 days at appropriate ratios (typically 1:3, 1:6, 1:12).

### 3.9. Cell Viability Assay

To assess the potential cytotoxic effect of SCE, a cell viability study was performed according to the previously described protocol [8]. Briefly, 10^4^ Caco-2 cells were seeded in 96-well plates, and after overnight incubation at 37 °C, treated with increasing concentrations of SCE (0.25–1 mg/mL) solubilized in cell culture medium just before the experiment. After 2 h of treatment, solutions were aspirated from the wells, and the cells were washed with pre-warmed PBS. Finally, 3-(4,5-dimethylthiazol-2-yl)-2,5-diphenyltetrazolium bromide (MTT) was used to assess cell viability as previously described [8]. Cell viability was expressed as a percentage of the absorbance intensity of untreated cells (controls) taken as 100%. To ensure that SCE did not interfere with the assay, the signal from the cell-free wells was subtracted from that of the treated cell well one. To supplement MTT data and support the hypothesis of no toxic effect due to treatment with SCE, cytotoxicity assays were carried out by careful examination of cells under a microscope, as indicated in USP 28 (United States Pharmacopeia edition 2005) as an alternative method [39]. This analysis was conducted by experienced personnel and photographs of the effects of SCE on cells are shown in the Supplementary Materials (Figures S2 and S3). Furthermore, it was mandatory to identify the most suitable drug concentration that would avoid cytotoxic effects on Caco-2 cells (a condition that may bias the permeability study) and that would allow the optimal chromatographic response when analyzing the samples obtained. Therefore, cells were exposed to increasing concentrations of drugs (1–10–100 µM) for 2 h. Concentrations were chosen to identify the best one to use for permeability studies that may ensure an adequate amount of compound to be quantified by the HPLC-UV/MS method, whilst not causing cytotoxic effects that would alter the experiment. In order to fully reproduce the conditions of the permeability studies and assess the possible induction of irreversible cytotoxic effects by SCE or drugs, cell viability was also checked after a further 24 h of incubation in free medium (2 h + 24 h). Cell viability was assessed as above described.

### 3.10. Caco-2 Cell Colture for Permeability Studies

Caco-2 cells were seeded at a density of 2 × 10^4^ on Transwell supports (0.4 µm pore polycarbonate membrane, 6.5 mm) and incubated overnight in a controlled atmosphere of 37 °C and 5% of CO_2_. The following day, the medium was changed to remove floating cells and fresh pre-warmed DMEM 20% was added (300 µL in the apical compartment and 1200 µL in the basal one). Cells were cultured for approximately 18–21 days in order to ensure that the cellular monolayers differentiated and many transport proteins and brush border hydrolases were expressed. Media were changed every 2–3 days.

### 3.11. Measurement of Cell Monolayer Integrity with Lucifer Yellow

The fluorescent probe Lucifer yellow (LY) was used to evaluate the integrity of the cellular monolayers after 18–21 days after seeding. Caco-2 cells were washed with Hanks’ Balanced Salt solution (HBSS) and treated with a solution 0.1 mg/mL of LY in pre-warmed HBSS for 60 min in the dark at 37 °C. Then, 150 µL from the basal wells were transferred to a black 96-well plate and the fluorescence intensity was measured at the excitation wavelength of 485 nm and an emission one of 535 nm (SYNERGY HTX reader, BioTek, Winooski, VT, USA). The results, expressed as a percentage of LY passing through the cell monolayer, were obtained according to the following equation:

% LY permeability = (sample-blank)/(LY-blank) × 100(4)

Measurement of cell monolayer integrity was performed on Caco-2 cells prior to any permeability experiments. To evaluate how sweet cherry extract may or may not affect cell monolayer integrity, LY permeability tests were conducted before (0 h) and during (2 h) the treatment with SCE. To evaluate the ability of the cellular monolayer to fully restore the membrane integrity, the LY permeability assay was also conducted after 24 h of treatment removal and incubating cells with fresh DMEM 20% for further 24 h (2 h + 24 h medium).

### 3.12. Permeability Studies in the Presence and Absence of SCE

For permeability studies, stock solutions of compounds were prepared by solubilizing 1.0 mg of accurately weighed powder with dimethyl sulfoxide (DMSO) and conserved at −20 °C. Immediately prior to the experiments, working solutions (WS) were prepared by diluting the stock solutions with pre-warmed HBSS to the final concentration of 10 µM. The final concentration of DMSO used never exceeded 0.1% *v*/*v*. The assays were performed in both the absorptive (from apical-to-basolateral compartment (A-B)) and the secretory direction (from basolateral-to-apical compartment (B-A)), both in the absence (Free) and presence of sweet cherry extract (+SCE [1 mg/mL]) diluted in HBSS. The final volumes used for the experiment were 300 µL in the apical compartment and 1200 µL in the basolateral one. Plates were incubated at 37 °C and maintained under gentle shaking (500 rpm) to reduce the effect of the unstirred water layer. At selected time points (T_0_–0.25–0.5–1–2 h-T_END_ for the A-B studies; T_0_–2 h–T_END_ for the B-A studies), samples (200 µL) were collected from the receiving compartments (from the basolateral compartment for the A-B assay; from the apical one for the B-A study) and replaced with the same volume of HBSS. The HPLC-UV/MS method was developed to quantify the amount of drug transferred across the cellular monolayer, and permeability (P_app_, 10^−6^ cm/s) and efflux ratio (ER) were calculated according to the following equations:

P_app_ = (dQ/dt) × 1/(A × C_0_) (5)

where dQ/dt is the steady-state flux (µmol s^−1^), A is the area of the filter (cm^2^), and C_0_ is the initial concentration in the donor compartment (µM).

ER = (P_app_ B-A)/(P_app_ A-B) (6)

### 3.13. Caco-2 Monolayer Permeability to Albumin

Due to its large molecular size, human serum albumin (HSA) was used as a probe to conduct experiments focusing on the dimensions of drug cargoes. Caco-2 cells were seeded as described above (see Section 3.10), and after 18–21 days, cells were exposed to 1 mg/mL HBSS solution of HSA. The permeability experiment was conducted in the absorptive direction (from apical-to-basolateral direction (A-B)), first in the absence of the extract (Free), and then in the presence of SCE in HBSS (+SCE [1 mg/mL]). Plates were incubated at 37 °C and maintained under gentle shaking (500 rpm). After 2 h of incubation with HSA, samples (200 µL) were collected from the receiving basolateral compartments and replaced with the same volume of HBSS. Finally, the amount of HSA that passed across the Caco-2 monolayer was quantified using the QuantiPro™ BCA Assay Kit (Sigma Aldrich S.r.l., Milan, Italy) with few modifications of the manufacturer’s protocol. Because of the possible complexing phaenomena between the cherry extract and HSA, primarily via hydrogen bonds and van der Waals forces, the quantification of HSA in the absence and presence of SCE was performed using specific calibration curves. In the case of HSA-free incubation, HSA stock solutions were prepared in HBSS and accurately diluted with the QuantiPro Working Reagent (QuantiPro WR, Sigma Aldrich, St. Louis, MI, USA). When in the presence of SCE, the quantification of HSA required the use of a specific calibration curve prepared with SCE; indeed, HSA-SCE (1:1 *v*/*v*) stock solutions were made in HBSS and appropriately diluted with QuantiPro WR until final concentrations reported in the protocol. Absorbance was quantified at 562 nm using a Multiskan SkyHigh Microplate Spectrophotometer (ThermoFisher, Waltham, MA, USA) after incubation for 2 h at 37 °C.

### 3.14. HPLC-UV/MS Method for Quantification of Tested Compounds

Chromatographic analyses were carried out using an Agilent 1260 Infinity HPLC-DAD system connected to an Agilent MSD 6130 system (Agilent Technologies, Palo Alto, CA, USA). At room temperature, a Kinetex C18-100 Å column (150 mm 4.6 mm, purchased from Phenomenex, Torrance, CA, USA) with a 5 µm particle size and gradient elution with a binary solution (eluent A: H_2_O, eluent B: ACN, both acidified with 0.1% *v*/*v* formic acid) was for the chromatographic separation. Analyses started with 5% B (from t = 0 to t = 1 min), then increased to 95% (from t = 1 to t = 10 min), then maintained at 95% (from t = 10 to t = 15 min), and finally returned to 5% eluent A in 1.0 min. The volume injected was 10 µL, and the flow rate 600 µL/min. UV detection was monitored at 254 nm, and spectra were collected in both the positive and negative modes within the scan range of *m*/*z* 100–1500.

### 3.15. Statistical Analysis

Statistical analysis was performed using GraphPad Prism 8.2 (GraphPad Software, La Jolla, CA, USA). Data are presented as the mean ± standard deviation (SD) of at least three independent experiments. The analysis of total phenol content, total anthocyanins content, and antioxidant activity were accomplished using one-way analysis of variance (ANOVA), and the means were compared using the Tukey post hoc test. Statistical analysis was performed using two-way ANOVA followed by Tukey’s multiple comparison test (cytotoxic effect of compounds on Caco-2 cells), one-way ANOVA followed by Dunnett’s multiple comparisons test (Lucifer Yellow permeability test), and Student’s *t*-test for unpaired samples (Caco-2 permeability assays and efflux ratio data in the absence and presence of SCE, and Molecule cargoes’ size experiment) as appropriate.

## 4. Conclusions

Although the poor permeability of the gastrointestinal tract limits the oral absorption of most compounds, including anticancer drugs, patients still prefer oral therapies to injections. Therefore, we focused our attention on the search for a non-toxic, effective, and reversible permeation enhancer capable of facilitating the oral absorption of anticancer compounds. We selected sweet cherry to prepare a polyphenol-enriched hydroalcoholic extract and investigate the ability of the extract to increase the permeability across the Caco-2 monolayer and consequently modify the efflux ratio of selected compounds. According to their oral bioavailability data, we selected atenolol, propranolol, and dasatinib as reference compounds and some pyrazolo[3,4-*d*]pyrimidine compounds from our internal library. In this second case, the selection was carried out thoroughly, taking into account the ADME properties. The extract was characterized by HPLC-UV-MS analysis, anthocyanins, flavonols, as well as some chlorogenic acid derivatives that were identified (see Table 2 and Figure S1) and analyzed in terms of antioxidant activity (ABTS and DPPH assays are 211.74 and 48.65 µmol of TE/g DE, respectively), total phenolic (TPC in the cherry extract is 8.44 mg GAE/g DE), and anthocyanins content (TAC of the cherry extract is 1.80 mg Cy-3-glu/g DE). Having verified the non-toxic effect of SCE on Caco-2 cells at increasing concentrations, we selected 1 mg/mL for further permeability studies (Figures S2 and S3 in the Supplementary Materials). First, we tested the efficacy of SCE in acting as a permeation enhancer by monitoring the passage of Lucifer yellow across the Caco-2 cell monolayer. The amount of the dye passed across the Caco-2 monolayer was more than five-fold enhanced and, most importantly, the initial permeability was almost completely restored after 24 h of extract removal (Figure 3). Cytotoxicity assays of the compounds were conducted on Caco-2 cells to select the appropriate concentration for further studies; the middle concentration of 10 µM was chosen for permeability studies that were performed both in the absence (Free) and in presence of SCE (+SCE [1 mg/mL]). As reported in Table 4 and Table 6, both reference and pyrazolo[3,4-*d*]pyrimidine compounds underwent a general and significant increase in permeability, often accompanied by an important decrease in efflux ratio (Table 5 and Table 7). Finally, in order to exclude the formation of excessively large gaps in the Caco-2 monolayer that could allow the passage of pathogens or xenobiotics, we investigated the size of the molecular cargo across the monolayer, taking advantage of the large molecular size of HSA. These results are particularly encouraging as they demonstrate that the permeation-promoting activity of SCE did not over-permeate the Caco-2 monolayer and prevent large cargoes from passing through. Although further investigations are required to better characterize the sweet cherry extract, explore the mechanism of action, and assess the in vivo efficacy and safety, our present findings demonstrate that polyphenol-rich sweet cherry extract is a highly effective oral drug uptake enhancer that may pave the way for novel oral pharmacological therapies.

## Figures and Tables

**Table 1:** Chemical structures and ADME data of pyrazolo[3,4-*d*]pyrimidine derivatives **1**–**7** previously published by our research group.

** 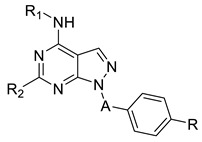 **					**ADME Data**		
**Compounds**	**R**	**A**	**R_1_**	**R_2_**	**Water ** **Solubility ** **(µg/mL)**	**PAMPA P_app_ 10^−6^ cm/s** **(MR %) ^d^**	**Metabolic** **Stability (%)**
**1 ^a^**	F	CH_2_CH(Cl)	CH_2_C_6_H_5_	H	-	10.7 (9.2)	84.2
**2 ^a^**	H	CH_2_CH(Cl)	CH_2_CH_2_C_6_H_5_	SCH_3_	-	3.2 (42.7)	96.6
**3 ^b^**	H	CH_2_CH(Cl)	C_6_H_3_-*o*Cl-*m*OH	H	0.053	4.3 (-)	86.3
**4 ^c^**	H	CH_2_CH(CH_3_)	C_6_H_4_-*m*OH	NHCH_2_CH_2_OH	134.2	0.2 (1)	99.4
**5 ^b^**	H	CH_2_CH(CH_3_)	C_6_H_3_-*o*Cl-*m*OH	SCH_2_CH_2_-4- morpholino	0.031	11.2 (6.6)	97.9
**6 ^b^**	H	CH_2_CH(CH_3_)	C_6_H_3_-*o*Cl-*m*OH	NHCH_2_CH_2_OH	0.103	0.1 (4.0)	97.9
**7 ^c^**	H	CH=CH	C_6_H_3_-*o*Cl-*m*OH	SCH_2_CH_2_-4- morpholino	0.036	5.5 (5.4)	97.6

^a^ Ref. [8]. ^b^ Ref. [9]. ^c^ Ref. [10]. ^d^ Membrane retention %. Empty cells were not determined.

**Table 2:** HPLC-UV/MS (ESI +/−) characterization of flavonoids and chlorogenic acid derivatives in sweet cherry extract.

Peak	Compound	RT ^a^ (min)	λ_max_^b^ (nm)	[M]^+ c^ (*m*/*z*)	[M-H]^− c^ (*m*/*z*)
**1**	Procyanidin B_1_	16.01	280	-	577
**2**	Cyanidin-3-O-rutinoside	16.89	520	595	-
**3**	Pelargonidin-3-O-rutinoside	17.33	520	579	-
**4**	*trans*-3-O-caffeoylquinic acid	17.58	330	-	353
**5**	Peonidin-3-O-rutinoside	18.04	520	609	-
**6**	*trans*-5-O-caffeoylquinic acid	18.55	330	-	353
**7**	*trans*-4-O-caffeoylquinic acid	18.74	330	-	353
**8**	*cis*-5-O-caffeoylquinic acid	18.95	320	-	353
**9**	Quercetin-3-O-rutinoside-7-O-glucoside	19.07	340	-	771
**10**	Quercetin-3-O-galactosyl-rhamnoside	20.04	-	-	609
**11**	Quercetin-3-O-rutinoside	20.18	360	-	609
**12**	*trans*,*trans*-3,5-di-O-caffeoylquinic acid	21.14	330	-	515
**13**	Kaempferol-3-O-rutinoside	21.62	360	-	593

^a^ Retention time. ^b^ Maximum wavelength of absorption. ^c^ The detection was conducted both in positive [M]^+^ and in negative [M-H]^−^ modes of ionization.

**Table 3:** Analysis of total phenols content (TPC), total anthocyanins content (TAC), and antioxidant activity (ABTS and DPPH) of cherry extract expressed as mean ± SD ^a^.

	TPC	TAC	Antioxidant Activity	
			ABTS	DPPH
Sweet cherry extract	8.44 ± 0.16	1.80 ± 0.09	211.74 ± 3.90	48.65 ± 2.44

^a^ Results represent the mean ± SD of three experiments. TPC is expressed as mg of gallic acid (GA) per gram of dry extract; TAC is expressed as milligrams of cyanidin-3-glucoside equivalent per g of dry extract. ABTS and DPPH are expressed as µmol of Trolox equivalent per gram of dry extract.

**Table 4:** Bidirectional permeability measured in Caco-2 cells for reference compounds atenolol, propranolol, and dasatinib.

Cpd	P_app_ A-B ^a^		P_app_ B-A ^a^	
	Free	+SCE [1 mg/mL]	Free	+SCE [1 mg/mL]
Atenolol	0.284 ± 0.011	9.112 ± 0.572	1.023 ± 0.104	7.273 ± 0.932
Propranolol	41.320 ± 4.280	93.071 ± 3.844	33.591 ± 0.967	37.156 ± 4.734
Dasatinib	2.023 ± 0.103	2.411 ± 0.120	36.858 ± 2.954	31.113 ± 0.342

^a^ Apparent permeability (P_app_) reported in cm/s × 10^−6^. ^b^ Efflux ratio calculated as the ratio of the permeability in the secretory direction (P_app_ B-A) and that in the absorptive direction (P_app_ A-B). Values are the means ± S.D. of *n* = 3 experiments run in triplicate.

**Table 5:** Efflux ratio calculated in Caco-2 cells for reference compounds atenolol, propranolol, and dasatinib.

	Reference Compounds		
	Atenolol	Propranolol	Dasatinib
Free	3.595 ± 0.231	0.819 ± 0.108	18.207 ± 0.529
+SCE [1 mg/mL]	0.803 ± 0.153	0.399 ± 0.034	12.926 ± 0.786

Efflux ratio (ER) calculated as the ratio of the permeability in the secretory direction (P_app_ B-A) and that one in the absorptive direction (P_app_ A-B). Values are the means ± S.D. of *n* = 3 experiments run in triplicate.

**Table 6:** Bidirectional permeability measured in Caco-2 cells for selected pyrazolo[3,4-*d*]pyrimidine derivatives **1**–**7**.

Cpd	P_app_ A-B ^a^		P_app_ B-A ^a^	
	Free	+SCE [1 mg/mL]	Free	+SCE [1 mg/mL]
1	4.943 ± 0.044	16.167 ± 0.014	15.111 ± 1.569	26.782 ± 3.681
2	0.110 ± 0.004	0.224 ± 0.009	10.412 ± 0.363	2.297 ± 0.279
3	1.680 ± 0.061	16.252 ± 0.537	4.744 ± 0.097	32.461 ± 0.784
4	5.108 ± 0.385	16.903 ± 0.743	34.513 ± 0.817	53.285 ± 1.802
5	1.249 ± 0.122	1.543 ± 0.095	8.041 ± 0.183	5.985 ± 0.286
6	3.323 ± 0.041	14.882 ± 1.864	18.980 ± 2.153	51.453 ± 0.118
7	0.477 ± 0.048	1.172 ± 0.120	0.229 ± 0.023	0.457 ± 0.108

^a^ Apparent permeability (P_app_) reported in cm/s × 10^−6^. Values are the means ± SD of *n* = 3 experiments run in triplicate.

**Table 7:** Efflux ratio calculated in Caco-2 cells for selected pyrazolo[3,4-*d*]pyrimidine derivatives **1**–**7**.

	Pyrazolo[3,4-*d*]pyrimidine Compounds						
	1	2	3	4	5	6	7
Free	3.056 ± 0.290	97.256 ± 4.489	2.826 ± 0.160	6.769 ± 0.351	6.462 ± 0.486	5.708 ± 0.578	0.481 ± 0.049
+SCE [1 mg/mL]	1.656 ± 0.226	10.216 ± 0.837	1.999 ± 0.114	3.158 ± 0.245	3.879 ± 0.054	3.485 ± 0.445	0.387 ± 0.052

Efflux ratio (ER) calculated as the ratio of the permeability in the secretory direction (P_app_ B-A) and that one in the absorptive direction (P_app_ A-B). Values are the means ± SD of *n* = *3* experiments run in triplicate.

## Data Availability

All the data are presented in this paper.

## Supplementary Materials

The following supporting information can be downloaded at: https://www.mdpi.com/article/10.3390/ph16111527/s1, Chemistry; Scheme S1: Chemical structures of reference and pyrazolo-pyrimidine compounds **1**–**7**; Figure S1: HPLC chromatogram of the sweet cherry extract; Figure S2: Cell viability studies of Sweet Cherry Extract on Caco-2 cells; Figure S3: Effects of Sweet Cherry Extract on Caco-2 cell line.

## References

[1] Lamson N.G., Fein K.C., Gleeson J.P., Newby A.N., Xian S., Cochran K., Chaudhary N., Melamed J.R., Ball R.L., Suri K. (2022). The Strawberry-Derived Permeation Enhancer Pelargonidin Enables Oral Protein Delivery. Proc. Natl. Acad. Sci. USA.

[2] Eisenmann E.D., Talebi Z., Sparreboom A., Baker S.D. (2022). Boosting the Oral Bioavailability of Anticancer Drugs through Intentional Drug—Drug Interactions. Basic Clin. Pharmacol. Toxicol..

[3] Mathur P., Rawal S., Patel B., Patel M. (2019). Oral Delivery of Anticancer Agents Using Nanoparticulate Drug Delivery System. Curr. Drug Metab..

[4] Kim J.C., Park E.J., Na D.H. (2022). Gastrointestinal Permeation Enhancers for the Development of Oral Peptide Pharmaceuticals. Pharmaceuticals.

[5] Fein K.C., Lamson N.G., Whitehead K.A. (2017). Structure-Function Analysis of Phenylpiperazine Derivatives as Intestinal Permeation Enhancers. Pharm. Res..

[6] Calvani M., Pasha A., Favre C. (2020). Nutraceutical Boom in Cancer: Inside the Labyrinth of Reactive Oxygen Species. Int. J. Mol. Sci..

[7] Gonçalves A.C., Rodrigues M., Flores-Félix J.D., Campos G., Nunes A.R., Ribeiro A.B., Silva L.R., Alves G. (2022). Sweet Cherry Phenolics Revealed to Be Promising Agents in Inhibiting P-Glycoprotein Activity and Increasing Cellular Viability under Oxidative Stress Conditions: In Vitro and in Silico Study. J. Food Sci..

[8] Poggialini F., Vagaggini C., Brai A., Pasqualini C., Crespan E., Maga G., Perini C., Cabella N., Botta L., Musumeci F. (2023). Biological Evaluation and In Vitro Characterization of ADME Profile of In-House Pyrazolo[3,4-d]Pyrimidines as Dual Tyrosine Kinase Inhibitors Active against Glioblastoma Multiforme. Pharmaceutics.

[9] Molinari A., Fallacara A.L., Di Maria S., Zamperini C., Poggialini F., Musumeci F., Schenone S., Angelucci A., Colapietro A., Crespan E. (2018). Efficient Optimization of Pyrazolo[3,4-d]Pyrimidines Derivatives as c-Src Kinase Inhibitors in Neuroblastoma Treatment. Bioorganic Med. Chem. Lett..

[10] Di Maria S., Picarazzi F., Mori M., Cianciusi A., Carbone A., Crespan E., Perini C., Sabetta S., Deplano S., Poggialini F. (2022). Novel Pyrazolo[3,4-d]Pyrimidines as Dual Src/Bcr-Abl Kinase Inhibitors: Synthesis and Biological Evaluation for Chronic Myeloid Leukemia Treatment. Bioorg Chem..

[11] Vilas-Boas A.A., Campos D.A., Nunes C., Ribeiro S., Nunes J., Oliveira A., Pintado M. (2020). Polyphenol Extraction by Different Techniques for Valorisation of Non-Compliant Portuguese Sweet Cherries towards a Novel Antioxidant Extract. Sustainability.

[12] Clodoveo M.L., Crupi P., Muraglia M., Naeem M.Y., Tardugno R., Limongelli F., Corbo F. (2023). The Main Phenolic Compounds Responsible for the Antioxidant Capacity of Sweet Cherry (*Prunus Avium* L.) Pulp. LWT.

[13] Vauzour D., Rodriguez-Mateos A., Corona G., Oruna-Concha M.J., Spencer J.P.E. (2010). Polyphenols and Human Health: Prevention of Disease and Mechanisms of Action. Nutrients.

[14] El-Nashar H.A.S., Aly S.H., Ahmadi A., El-Shazly M. (2021). The Impact of Polyphenolics in the Management of Breast Cancer: Mechanistic Aspects and Recent Patents. Recent. Pat. Anticancer. Drug Discov..

[15] Manach C., Williamson G., Morand C., Scalbert A., Rémésy C. (2005). Bioavailability and Bioefficacy of Polyphenols in Humans. I. Review of 97 Bioavailability Studies 1–3. Am. J. Clin. Nutr..

[16] Gonçalves A., Bento C., Jesus F., Alves G., Silva L., Rahman A. (2018). Chapter 2—Sweet Cherry Phenolic Compounds: Identification, Characterization, and Health Benefits. Studies in Natural Products Chemistry.

[17] Özen M., Özdemir N., Ertekin Filiz B., Budak N.H., Kök-Taş T. (2020). Sour Cherry (*Prunus Cerasus* L.) Vinegars Produced from Fresh Fruit or Juice Concentrate: Bioactive Compounds, Volatile Aroma Compounds and Antioxidant Capacities. Food Chem..

[18] Vinson J.A., Su X., Zubik L., Bose P. (2001). Phenol Antioxidant Quantity and Quality in Foods: Fruits. J. Agric. Food Chem..

[19] Sokół-Łe̜towska A., Kucharska A.Z., Hodun G., Gołba M. (2020). Chemical Composition of 21 Cultivars of Sour Cherry (*Prunus Cerasus*) Fruit Cultivated in Poland. Molecules.

[20] Serrano M., Díaz-Mula H.M., Zapata P.J., Castillo S., Guillén F., Martínez-Romero D., Valverde J.M., Valero D. (2009). Maturity Stage at Harvest Determines the Fruit Quality and Antioxidant Potential after Storage of Sweet Cherry Cultivars. J. Agric. Food Chem..

[21] Ferretti G., Bacchetti T., Belleggia A., Neri D. (2010). Cherry Antioxidants: From Farm to Table. Molecules.

[22] Blando F., Gerardi C., Nicoletti I. (2004). Sour Cherry (Prunus Cerasus L) Anthocyanins as Ingredients for Functional Foods. J. Biomed. Biotechnol..

[23] Yazdanian M., Glynn L.S., Wright L.J., Hawi A. (1998). Correlating Partitioning and Caco-2 Cell Permeability of Structurally Diverse Small Molecular Weight Compounds. Pharm. Res..

[24] Honeywell R.J., Hitzerd S., Kathmann I., Peters G.J. (2016). Transport of Six Tyrosine Kinase Inhibitors: Active or Passive ?. ADMET DMPK.

[25] European Union European Medicines Agency. https://www.ema.europa.eu/en.

[26] Chen Y., Agarwal S., Shaik N.M., Chen C., Yang Z., Elmquist W.F. (2009). P-Glycoprotein and Breast Cancer Resistance Protein Influence Brain Distribution of Dasatinib. J. Pharmacol. Exp. Ther..

[27] Cunha R.D., Bae S., Murry D.J., An G. (2016). TKI Combination Therapy: Strategy to Enhance Dasatinib Uptake through Inhibiting Pgp- And BCRP- Mediated Efflux. Biopharm. Drug Dispos..

[28] Chen X., Slättengren T., Lange E.C.M., Smith D.E., Hammarlund-Udenaes M. (2017). Revisiting Atenolol as a Low Passive Permeability Marker. Fluids Barriers CNS.

[29] Yin J., Duan H., Shirasaka Y., Prasad B., Wang J. (2015). Atenolol Renal Secretion Is Mediated by Human Organic Cation Transporter 2 and Multidrug and Toxin Extrusion Proteins. Drug Metab. Dispos..

[30] Hayeshi R., Hilgendorf C., Artursson P., Augustijns P., Brodin B., Dehertogh P., Fisher K., Fossati L., Hovenkamp E., Korjamo T. (2008). Comparison of Drug Transporter Gene Expression and Functionality in Caco-2 Cells from 10 Different Laboratories. Eur. J. Pharm. Sci..

[31] Uchida M., Fukazawa T., Yamazaki Y., Hashimoto H., Miyamoto Y. (2009). A Modified Fast (4 Day) 96-Well Plate Caco-2 Permeability Assay. J. Pharmacol. Toxicol. Methods.

[32] Honeywell R.J., Kathmann I., Giovannetti E., Tibaldi C., Smit E.F., Rovithi M.N., Verheul H.M.W., Peters G.J. (2020). Epithelial Transfer of the Tyrosine Kinase Inhibitors Erlotinib, Gefitinib, Afatinib, Crizotinib, Sorafenib, Sunitinib, and Dasatinib: Implications for Clinical Resistance. Cancers.

[33] The International Trasporter Consortium (2010). Membrane Transporters in Drug Development. Nat. Rev. Drug Discov..

[34] Kim K.S., Jiang C., Kim J.Y., Park J.H., Kim H.R., Lee S.H., Kim H.S., Yoon S. (2020). Low-Dose Crizotinib, a Tyrosine Kinase Inhibitor, Highly and Specifically Sensitizes Chemoresistant Cancer Cells Through Induction of Late Apoptosis In Vivo and In Vitro. Front. Oncol..

[35] Shukla S., Chen Z.-S., Ambudkar S.V. (2012). Tyrosine Kinase Inhibitors as Modulators of ABC- Transporter Mediated Drug Resistance. Drug Resist. Updates.

[36] Fallacara A.L., Zamperini C., Podolski-Renic A., Dinic J., Stankovic T., Stepanovic M., Arianna M., Rango E., Iovenitti G., Molinari A. (2019). A New Strategy for Glioblastoma Treatment: In Vitroand In Vivo Preclinical Characterization of Si306,a Pyrazolo[3,4-d]Pyrimidine DualSrc/P-Glycoprotein Inhibitor. Cancers.

[37] Brai A., Poggialini F., Trivisani C., Tarchi F., Francardi V., Dreassi E. (2023). Efficient Use of Agricultural Waste to Naturally Fortify Tenebrio Molitor Mealworms and evaluation of Their Nutraceutical Properties. J. Insects Food Feed.

[38] Brai A., Vagaggini C., Pasqualini C., Poggialini F., Tarchi F., Francardi V., Dreassi E. (2023). Use of Distillery By-Products as Tenebrio Molitor Mealworm Feed Supplement. J. Insects Food Feed.

[39] Chiaino E., Micucci M., Budriesi R., Mattioli L.B., Marzetti C., Corsini M., Frosini M. (2021). Hibiscus Flower and Olive Leaf Extracts Activate Apoptosis in SH-SY5Y Cells. Antioxidants.

