# Supplementary material for: Sweet Cherry Extract as Permeation Enhancer of Tyrosine Kinase Inhibitors: A Promising Prospective for Future Oral Anticancer Therapies
Source: Pharmaceuticals (Basel). 2023 Oct 27;16(11):1527. doi: 10.3390/ph16111527 (PMC10674987; doi:10.3390/ph16111527)
Supplement: Supplementary file 1 [file pharmaceuticals-16-01527-s001.zip › pharmaceuticals-2661291-supplementary.pdf]

Supplementary Materials for:

Sweet cherry extract as permeation enhancer of tyrosine kinase inhibitors: a promising prospective for future oral anticancer therapies

Federica Poggialini [1], Chiara Vagaggini [1], Annalaura Brai [1], Claudia Pasqualini [1], Anna Carbone [2], Francesca Musumeci [2], Silvia Schenone [2], and Elena Dreassi [1,*]

[1] Department of Biotechnology, Chemistry, and Pharmacy (DBCF), University of Siena, 53100 Siena Italy; federic.poggialini@unisi.it (F.P.); chiara.vagaggini@student.unisi.it (C.V.); annalaura.brai@unisi.it (A.B.); pasqualini5@student.unisi.it (C.P.) elena.dreassi@unisi.it (E.D.)

[2] Department of Pharmacy, University of Genoa, 16132 Genoa, Italy; anna.carbone1@unige.it (A.C.), francesca.musumeci@unige.it (F.M.), silvia.schenone@unige.it (S.S.)

* Correspondence: elena.dreassi@unisi.it; Tel.: +39-0577-234321

Summary

Chemistry 2

Scheme S1. Chemical structures of reference and pyrazolo-pyrimidine compounds 1-7.................... 4

Figure S1. HPLC chromatogram of the sweet cherry extract. 5

Figure S2. Cell viability studies of Sweet Cherry Extract on Caco-2 cells. 6

Figure S3 Effects of Sweet Cherry Extract on Caco-2 cell line. 7

Chemistry

N-Benzyl-1-[2-chloro-2-(4-fluorophenyl)ethyl]-1H-pyrazolo[3,4-d]pyrimidin-4-amine (1). White solid, mp 143-144 °C, yield 79%. ^{1}H NMR: δ 4.62-4.95 (m, 4H, CH_2N + CH_2Ar), 5.40-5.51 (m, 1H, CHCl), 6.96-7.40 (m, 9H Ar), 7.77 (s, 1H, H-3), 8.28 (s, 1H, H-6). IR cm^{-1}: 3198 (NH). Anal. ($C_{20}H_{17}N_5ClF$) C, H, N [8].

1-(2-chloro-2-phenylethyl)-6-methylthio-N-phenethyl-1H-pyrazolo[3,4-d]pyrimidin-4-amine (2). White solid, mp 73–74 °C, yield 76%. ^{1}H NMR: δ 2.59 (s, 3H, CH_3S), 2.98 (q, J = 6.0, 2H, $CH_2C_6H_5$), 3.87 (q, J = 6.0, 2H, CH_2NH), 4.70–4.95 (m, 2H, CH_2N), 5.30 (br s, 1H, NH, disappears with D_2O), 5.50–5.60 (m, 1H, CHCl), 7.19–7.48 (m, 10H Ar), 7.73 (s, 1H, H-3). IR cm^{-1}: 3445 (NH) [9].

4-Chloro-3-{[1-(2-chloro-2-phenylethyl)-1H-pyrazolo[3,4-d]pyrimidin-4-yl]amino}phenol (3). Yield: 76%. 1H-NMR (Acetone-d6) δ (ppm): 8.77 (bs, 2H); 8.34 (s, 1H); 7.80 (s, 1H); 7.52-7.50 (m, 3H); 7.37-7.30 (m, 4H); 6.80 (m, 1H); 5.66 (t, J = 8Hz, 1H); 5.01 (dd, J = 7Hz, 1H); 4.83 (dd, J = 7Hz, 1H); 1.40 (m, 3H); 13C NMR (Acetone-d6) δ (ppm): 156.8, 155.7, 155.4, 154.4, 138.4, 135.9, 131.8, 131.7 130.1, 129.9, 128.9, 127.5, 118.8, 114.5, 114.3, 100.1, 60.35, 53.2. HRMS (ESI) m/z calcd for $C_{19}H_{15}C_{12}N_5O$ $[M+H]^+$ 400.0653 found 400.0648 [9].

3-{[6-[(2-Hydroxyethyl)amino]-1-(2-phenylpropyl)-1H-pyrazolo[3,4-d]pyrimidin-4-yl]amino}phenol (4). Yield: 80% over two steps. 1H-NMR (CD_3OD) δ (ppm): 7.80 (s, 1H); 7-38 (m, 1H); 7.23-7.20 (m, 4H); 7.14-7.10 (m, 3H); 6.54 (m, 1H); 4.28 (m, 2H); 3.74 (t, J = 5Hz, 2H); 3.56 (t, J = 5Hz, 2H); 3.45 (m, 1H); 1.22 (m, 3H). 13C NMR (CDCl3) δ (ppm): 161.8, 157.4, 155.8, 155.4, 143.5, 140.4, 131.7, 129.1, 128.1, 126.9, 126.3, 112.7, 110.4, 108.7, 96.1, 61.3, 52.7, 43.7, 39.8, 17.7. HRMS (ESI) m/z calcd for $C_{22}H_{24}N_6O_2$ $[M+H]^+$ 405.1960 found 405.1969 [9].

4-Chloro-3-{[6-[(2-morpholinoethyl)thio]-1-(2-phenylpropyl)-1H-pyrazolo[3,4-d]pyrimidin-4-yl]amino}phenol (5). Yield: 68%. 1H-NMR ($CDCl_3$) δ (ppm): 8.46 (bs, 2H); 7.91 (s, 1H); 7.57 (s, 1H); 7.29-7.17 (m, 5H); 6.59 (d, J = 7Hz); 4.46 (m, 2H); 3.84 (m, 4H); 3.55 (m, 1H); 3.40 (m, 2H); 2.94 (m, 2H); 2.68 (m, 4H); 1.25 (m, 3H). 13C NMR ($CDCl_3$) δ (ppm): 167.4, 156.3, 154.7, 143.2, 134.9, 129.9, 129.6, 128.5, 127.3, 126.8, 111.8, 109.1, 99.5, 65.3, 59.9, 53.7, 39.9, 29.7, 27.1, 18.8. HRMS (ESI) m/z calcd for $C_{26}H_{29}ClN_6O_2S$ $[M+H]^+$ 525.1758 found 525.1750 [9].

4-Chloro-3-{[6-[(2-hydroxyethyl)amino]-1-(2-phenylpropyl)-1H-pyrazolo[3,4-d]pyrimidin-4-yl]amino}phenol (6). Yield: 65% over two steps. 1H-NMR (CD_3OD) δ (ppm): 7.50 (s, 1H); 7.29-7.12 (m, 7H); 6.69 (d, J =8Hz, 1H); 4.30 (m, 2H); 3.72 (m, 2H); 3.51 (m, 2H); 3.31 (m, 1H); 1.22 (m, 3H); 13C NMR ($CDCl_3$) δ (ppm): 161.7, 156.6, 156.2, 156, 143.4, 136, 131.6, 129.7, 128.2, 126.8, 126.3, 119.2, 114.4, 113.8, 95.7, 61.3, 52.7, 43.6, 39.8, 39, 17.8. HRMS (ESI) m/z calcd for $C_{22}H_{23}ClN_6O_2$ $[M+H]^+$ 439.1561 found 439.1563 [9].

(E)-4-chloro-3-((6-((2-morpholinoethyl)thio)-1-styryl-1H-pyrazolo[3,4-d]pyrimidin-4-yl)amino)phenol (7). Yield: 67%. M.p.: 189-191 °C. 1H NMR (400 MHz, CDCl3): δ ppm 8.42 (bs, 1H), 8.03 (s, 1H), 7.94 (d, Jtrans = 15.0 Hz, 1H), 7.65 (s, 1H), 7.54-7.48 (m, 3H), 7.35 (d, Jtrans = 15.0 Hz, 1H), 7.31-7.26 (m, 4H), 6.65-6.60 (m, 1H), 3.90-3.86 (m, 4H), 3.44-3.40 (m, 2H), 3.96-3.92 (m, 2H), 2.70-2.67 (m, 4H). 13C NMR (400 MHz, CDCl3): δ ppm 172.01, 158.42, 157.64, 154.02, 138.71, 134.89, 132.23, 133.56, 129.31, 128.62, 128.59, 128.50, 127.91, 121.82, 107.3, 102.45, 100.56, 67.81, 57.45, 56.81, 29.90. MS: 508.7 m/z $[M]^-$ [10].

Scheme S1. Chemical structures of reference and pyrazolo-pyrimidine compounds 1-7

Atenolol	Propranolol
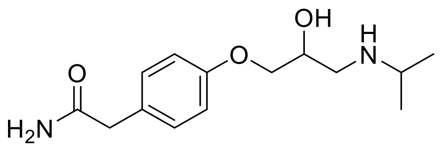	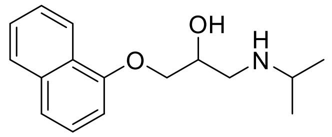

Dasatinib	Compound 1
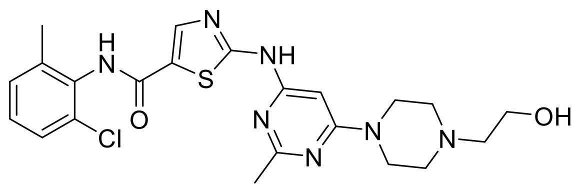	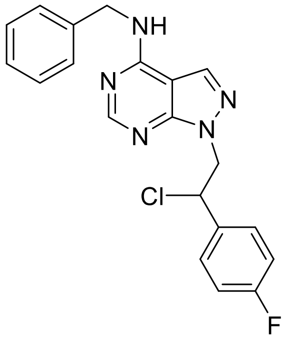

Compound 2	Compound 3	Compound 4
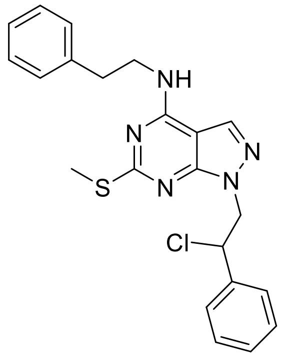	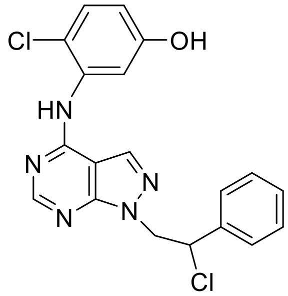	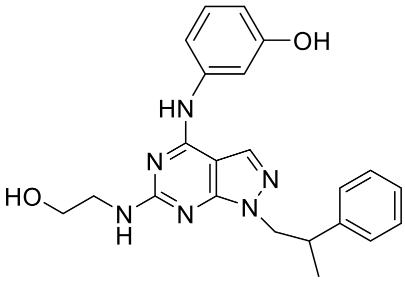

Compound 5	Compound 6	Compound 7
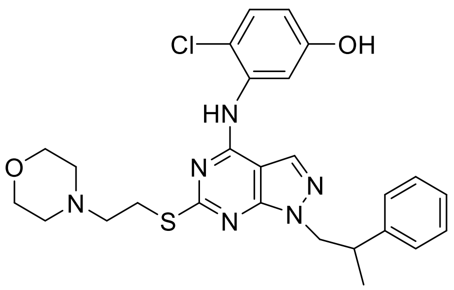	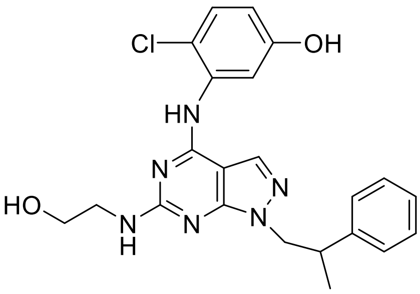	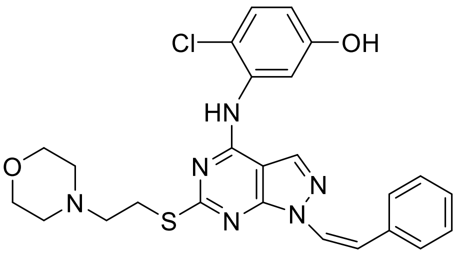

Figure S1. HPLC chromatogram of the sweet cherry extract.

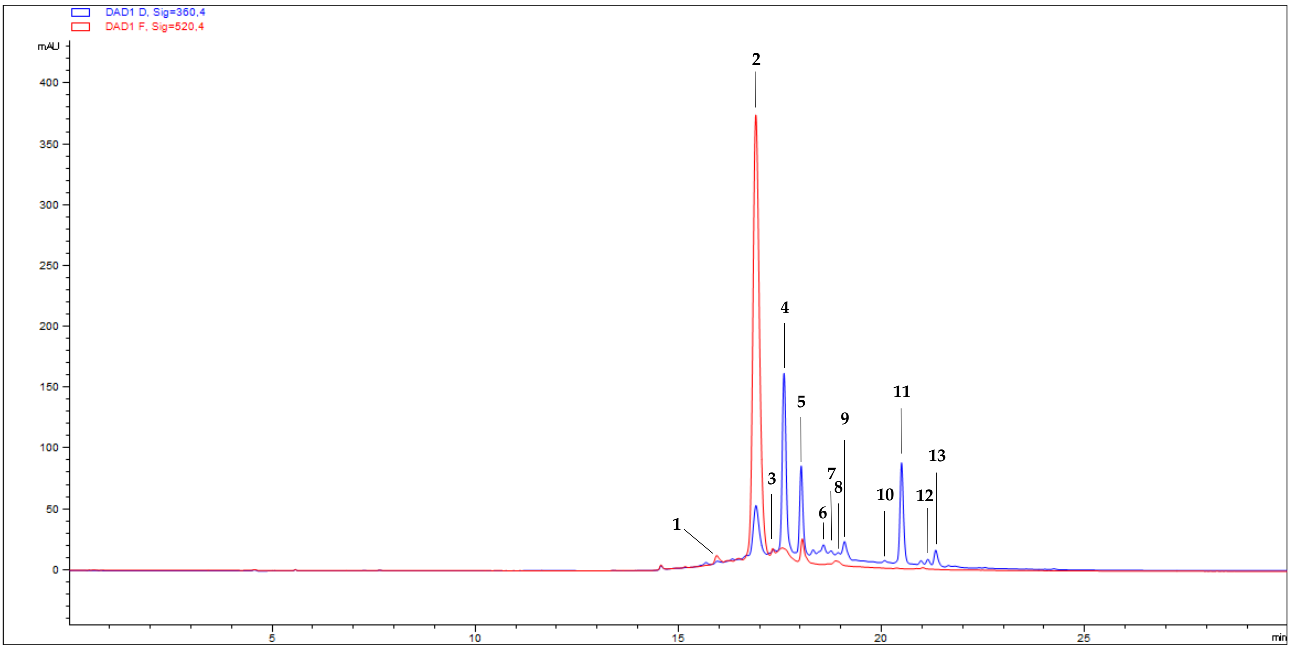

Figure S1. HPLC chromatogram obtained for the sweet cherry extract (SCE) at wavelengths of 360 nm (blue) and 520 nm (red). Peak 1: Procyanidin B1. Peak 2: Cyanidin-3-O-rutinoside. Peak 3: Pelargonidin-3-O-rutinoside. Peak 4: *trans*-3-O-caffeoylquinic acid. Peak 5: Peonidin-3-O-rutinoside. Peak 6: *trans*-5-O-caffeoylquinic acid. Peak 7: *trans*-4-O-caffeoylquinic acid. Peak 8: *cis*-5-O-caffeoylquinic acid. Peak 9: Quercetin-3-O-rutinoside-7-O-glucoside. Peak 10: Quercetin-3-O-galactosyl-rhamnoside. Peak 11: Quercetin-3-O-rutinoside. Peak 12: *trans,trans*-3,5-di-O-caffeoylquinic acid. Peak 13: Kaempferol-3-O-rutinoside.

Figure S2. Cell viability studies of Sweet Cherry Extract on Caco-2 cells.

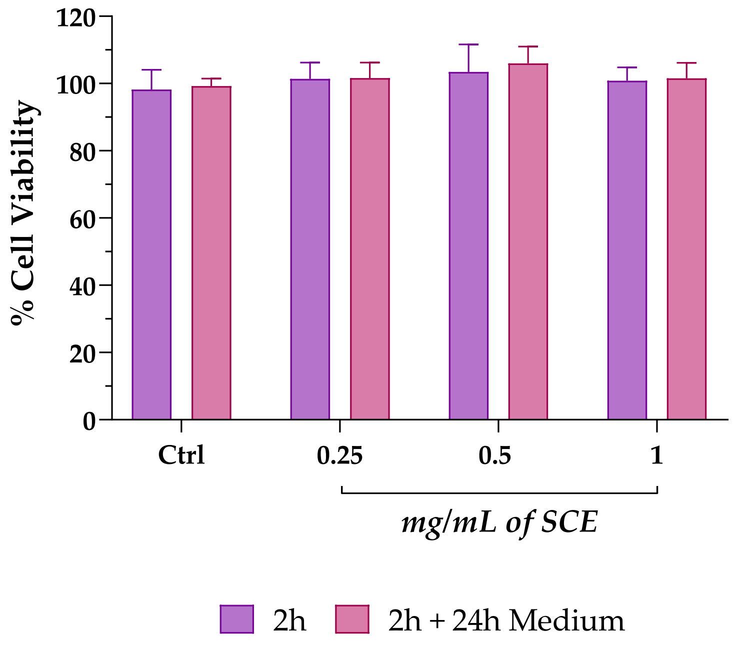

Figure S2. No-toxic effect of sweet cherry extract (SCE) on Caco-2 cell line. Cells were exposed to increasing concentration of the extract (from 0.25 mg/mL to 10 mg/mL in DMEM 20%) for 2h and then incubated for further 24h in the presence of fresh, SCE-free medium. Cell viability was determined by MTT assay.

Figure S3 Effects of Sweet Cherry Extract on Caco-2 cell line.

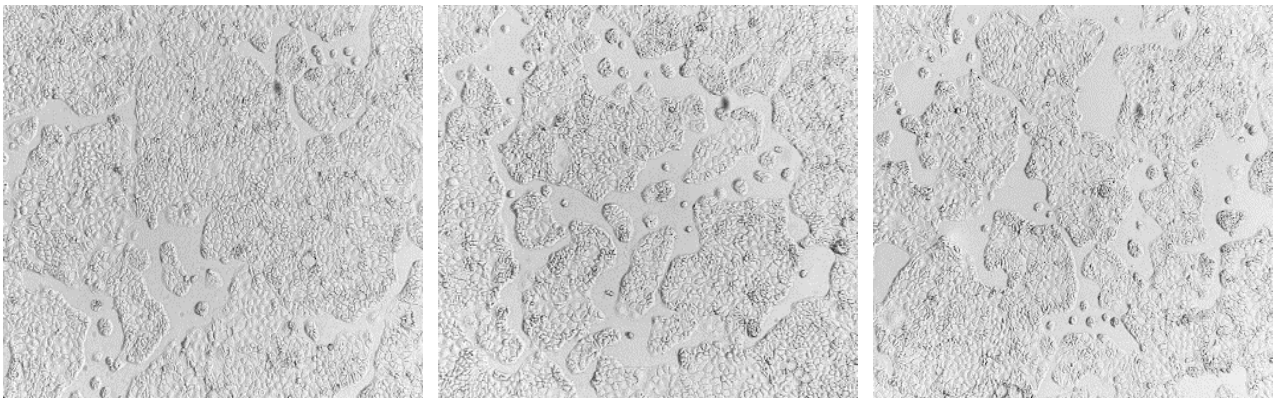

(**a**) (**b**) (c)

Figure S3. Morphological comparison performed at microscopy (scale bar 500 μm) between untreated Caco-2 cells (panel a), those treated with 1 mg/mL of sweet cherry extract for 2h (panel b) and those treated for 2 h and then incubated for other 24h with fresh-free medium (panel c). No significant morphological alterations, such as tendency to round-up, shrinkage, loss of contact with adjacent cells, membrane blebbing and formation of apoptotic bodies are evident.
